# Exome-assistant: a rapid and easy detection of disease-related genes and genetic variations from exome sequencing

**DOI:** 10.1186/1471-2164-13-692

**Published:** 2012-12-11

**Authors:** Qi Liu, Enjian Shen, Qingjie Min, Xueying Li, Xin Wang, Xianfeng Li, Zhong Sheng Sun, Jinyu Wu

**Affiliations:** 1 Institute of Genomic Medicine, Wenzhou Medical College, Wenzhou 325035, China; 2 Beijing Institutes for Biological Sciences, Chinese Academy of Science, Beijing 100101, China

**Keywords:** Next generation sequencing, Mendelian disease, Single nucleotide polymorphisms, Insertions and deletions, Variation filtering, Minor allele frequency

## Abstract

**Background:**

Protein-coding regions in human genes harbor 85% of the mutations that are associated with disease-related traits. Compared with whole-genome sequencing of complex samples, exome sequencing serves as an alternative option because of its dramatically reduced cost. In fact, exome sequencing has been successfully applied to identify the cause of several Mendelian disorders, such as Miller and Schinzel-Giedio syndrome. However, there remain great challenges in handling the huge data generated by exome sequencing and in identifying potential disease-related genetic variations.

**Results:**

In this study, Exome-assistant (http://122.228.158.106/exomeassistant), a convenient tool for submitting and annotating single nucleotide polymorphisms (SNPs) and insertion/deletion variations (InDels), was developed to rapidly detect candidate disease-related genetic variations from exome sequencing projects. Versatile filter criteria are provided by Exome-assistant to meet different users’ requirements. Exome-assistant consists of four modules: the single case module, the two cases module, the multiple cases module, and the reanalysis module. The two cases and multiple cases modules allow users to identify sample-specific and common variations. The multiple cases module also supports family-based studies and Mendelian filtering. The identified candidate disease-related genetic variations can be annotated according to their sample features.

**Conclusions:**

In summary, by exploring exome sequencing data, Exome-assistant can provide researchers with detailed biological insights into genetic variation events and permits the identification of potential genetic causes of human diseases and related traits.

## Background

Genome-wide genotyping has been very successful in elucidating the genetic basis of phenotypic traits, such as diseases [1]. One of the major goals of genotyping studies is to identify variants, especially disease-associated variants, such as single nucleotide polymorphisms (SNPs) and insertions and deletions (InDels). The advent of next-generation sequencing technologies is a great leap forward in DNA sequencing because of their capacity to generate massive amounts of data in a short time at low cost. Thus, they are very useful in genome-wide genotyping and associated studies [2]. Currently, however, whole genome sequencing of large numbers of individuals is still too expensive for many researchers. As an alternative, we employed exome sequencing, which targets the protein-coding regions, involved in approximately 85% of disease-causing mutations [3,4].

Indeed, exome sequencing has been successfully applied to the identification of allelic variants in the context of rare monogenic diseases. For example, exome sequencing was applied to a small number of unrelated, affected individuals and successfully identified a causative gene for Freeman-Sheldon syndrome [4]. Ng. et al. illustrated the utility of exome capture to search for variants causing Miller syndrome in three unrelated families [5]. Recently, using genome-wide linkage and whole exome sequencing, a homozygous missense mutation in the Fas-associated death domain protein (FADD) gene was found to be associated with autoimmune lymphoproliferative syndrome (ALPS).

However, the large amount of data generated by exome sequencing has still posed a bioinformatics challenge in mining the potential biological implications. Subsequently, several tools have become available for the detection of genetic variants from sequenced reads, such as Genome Analysis Tool-kit (GATK) [6], SAMtools [7], SOAPsnp [8], VarScan [9], SNVer [10], VAAST [11], SeqGene [12], Pindel [13] and BreakDancer [14]. The detected genetic variants need to be further annotated and require substantial bioinformatics experience to use them. Although some tools have been developed for the annotation of variations, such as WGAViewer [15], ANNOVAR [16], CandiSNPer [17], GAMES [18], SNPnexus [19], SeqAnt [20], TREAT [21], NGS-SNP [22], F-SNP [23], SNAP [24], variant tools [25], and VAR-MD [26], identifying of a small subset of functionally important sites from large amounts of detected variation data remains a challenge.

In this study, Exome-assistant was developed to provide the research community with a user-friendly and efficient tool for analyzing large amounts of genetic variants identified from exome sequencing studies. Exome-assistant provides researchers with detailed biological insights into genetic variation events and facilitates the identification of the potential genetic causes of human diseases and related traits from exome sequencing.

## Implementation

Exome-assistant consists of four modules: single case, two cases, multiple cases and re-analysis. The single case module is responsible for functional annotation and variations filtering. Two cases and multiple cases modules, which are based on the single case module, aim to identify the shared and unique variations between or among samples. In the multiple cases module, family-based study and Mendelian filtering can also be performed by Exome-assistant. The re-analysis module is designed to run previous submitted jobs with different parameters, which avoids resubmitting the sample data.

**Figure 1:**
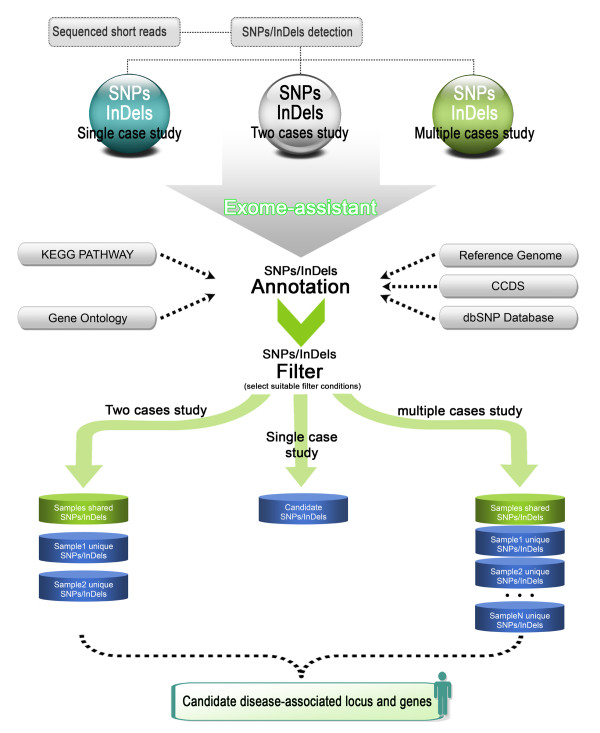
The workflow of the Exome-assistant pipeline.

The basic principle of the single case analysis is to annotate the SNPs/InDels based on the information from public databases (Figure 1), which include: CCDS [27] and dbSNP (v.137) [28] for site/region-specific annotation, KEGG pathway [29] and Gene Ontology for gene-based annotation. In the dbSNP database, the SNPs flagged as ‘clinically associated’ are considered as disease-related SNPs and excluded from the database. The human reference gene definition was downloaded from UCSC (http://hgdownload.cse.ucsc.edu) and integrated into Exome-assistant. Also integrated are the HapMap data, which are a resource of genotype data from ~4 million common SNPs derived from four human populations (African YRI, Japanese JPT, Han Chinese CHB and European CEU). It is used to calculate minor allele frequency (MAF) of SNPs in different populations and estimate whether a significant difference exists between the samples (p < 0.05) using a chi-square test. To better understand if the variations, especially non-synonymous variations, lead to functional alterations to the corresponding genes, the SIFT algorithm [30] was also integrated into Exome-assistant.

Exome-assistant first scans the reference genome and the CCDS database to perform site/region specific annotations, and classifies the variations into missense variations, nonsense variations, read-through variations and synonymous coding variations. In addition, the variations are also searched against dbSNP and those that are not present in dbSNP are assigned as novel SNPs/InDels. The novel variations, plus the known disease-associated variations, are then further classified as intergenic variations, intronic variations, 5^′^/3^′^-UTR variations and splice site variations, based on gene model definitions.

To explore the potential genetic variations involved in disease and traits, it is essential to reduce large numbers of variations into small subsets. The single case module provides users many criteria to filter the raw variation data. The criteria for filtering SNPs include: 1) whether the variation is novel or known to be disease-related; 2) whether the allele frequency is distinct from that in HapMap; 3) SNP mutation type; 4) SNP classification; and 5) impact of the SNP on the protein product. Meanwhile, the InDel filtering criteria include: 1) whether the InDel is novel or known to be disease-related; 2) whether the InDel is located in the coding region; 3) the mutation type of the InDel; and 4) the impact of InDel on the protein product.

Sample comparison, which aims to identify phenotype-associated variations, especially those associated with disease, is widely used in genetic studies, where the different annotated variations are scanned among or between samples. Exome-assistant includes two cases and multiple cases analysis. The primary motivation for developing the two cases module was to identify potential rare disease causal variations and genes, as well as to provide the ability to consider lower frequency gene disorders. In the multiple cases analyses, after filtering by single case module, the variations shared by disease samples are selected and those present in control samples (if they are submitted) are excluded from further analysis. In addition, the variations shared by disease samples, whose number can be custom-defined and must be minimum of two, are considered as potential disease-causing genetic variations. Moreover, family-based analysis can be performed, in which the candidate disease-related SNPs/InDels shared by family can be detected by setting the family trios. In addition, the Mendelian filtering parameters were added for users, which allows supporting the Mendilian filtering of SNPs/InDels in rare Mendelian disease families.

## Results and discussion

### Input

Exome-assistant supplies users with simple, user-friendly interface for analyzing extensively their variations from exome sequencing studies. The input of Exome-assistant is the SNPs/InDels calling results, which are generated by SNPs/InDels calling tools, such as SAMtools [7], SOAPsnp [8], GATK [6], BreakDancer [14], and Pindel [13]. Exome-assistant accepts text files as input (VCF, SOAPsnp, Pileup format for SNPs and VCF, Pileup format for InDels), in which each line represents one genetic variation. It takes ~0.5 hour to finish the analyses of one sample. Exome-assistant has a queuing module to control user-submitted jobs, where it executes two jobs in parallel and puts the remaining jobs into a queue. When the submission is finished, the server provides users with a job ID number, which can be used to retrieve the results once the job is finished or to reanalyze previously submitted data.

### Output

**Figure 2:**
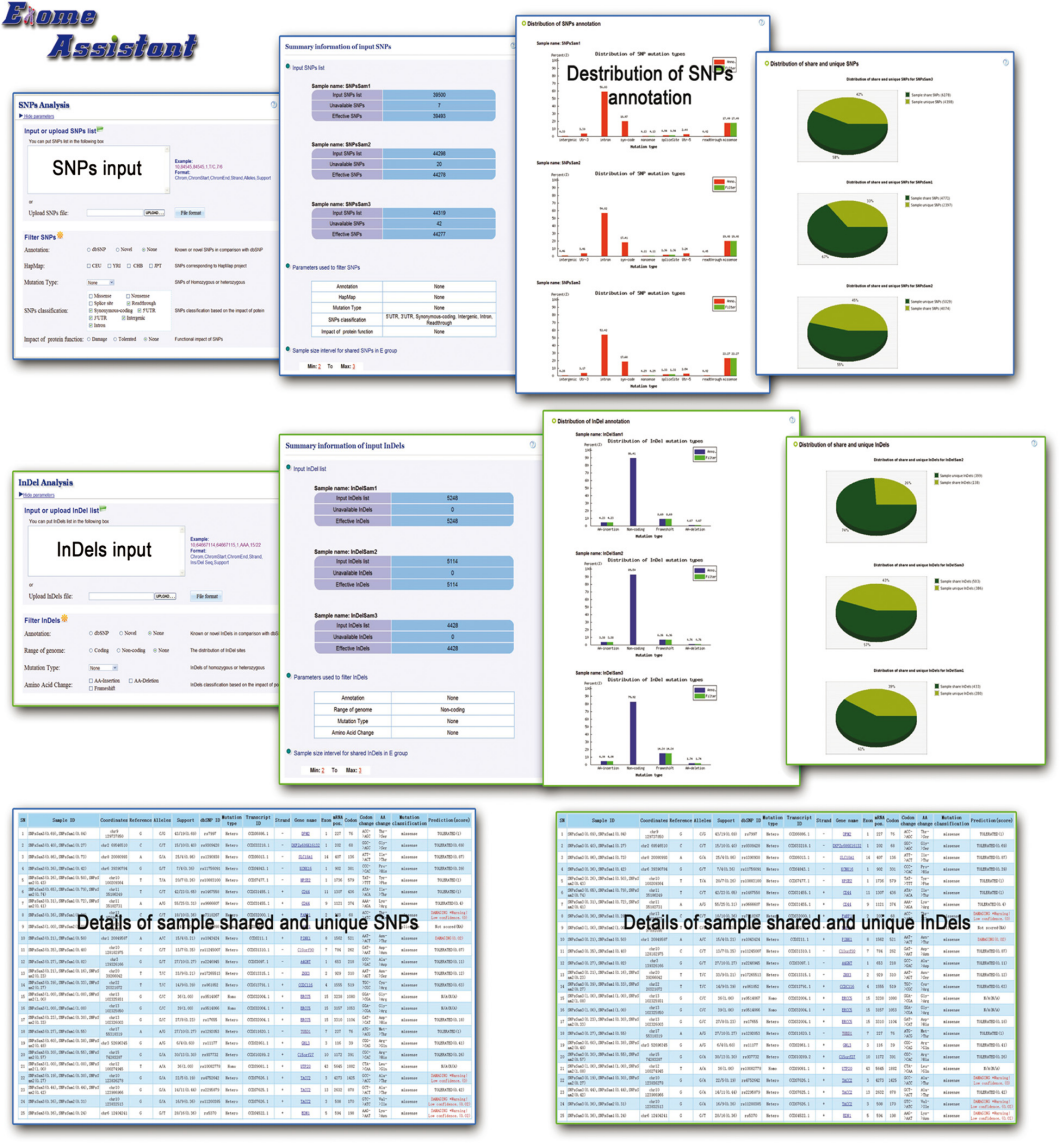
Screenshot of Exome-assistant.

Exome-assistant provides flexible and intuitive windows for convenient analysis and viewing of the results. In addition, all the results, including the input list, intermediate annotation results and the last results can be downloaded in a gzip-compressed format from the results webpage. A typical result of a single case analysis contains three parts for SNPs/InDels: the summary information of input variations, the distribution of variation annotation and the detailed annotation of each variation (Figure 2).

The summary information provides an overview of input variations, including the number of input variations, unavailable variations (erroneous variations records), effective variations, and the parameters set by user to filter the variations. The distributions of variations are shown in the form of a histogram and show the percentages of each category of variations before and after variation filtering. The categories consist of intergenic, UTR-3, intron, syn-code, nonsense, splice site, UTR-5, read through, missense for SNPs, and AA-insertion, Non-coding, Frame shift, an AA-deletion for InDels.

The detailed annotations of each variation are shown in a table format. The annotations of SNPs include dbSNP rs ID, gene name, transcript ID, amino-acid changes, frequency of SNP, mutation type, SNP functions (e.g. missense), prediction of functional damage to the protein product, etc. The annotations for InDels include SN (session number), coordinates on the chromosome, mutated strand, InDel sequence, substitution class, dbSNP ID, support frequency, gene name, and region of occurrence. In addition, links to other related databases, such as the gene annotation database, are also provided.

In two cases and multiple case analyses, apart from the above information for each sample, the summary information of comparison between samples describes the percentages of shared and unique variations between the samples. Meanwhile, the detailed annotations of shared and unique variations are shown in separate tables, which enable choosing variations for further study.

### Performance

We used Exome-assistant to identify and characterize disease-related genes in Familial amyotrophic lateral sclerosis (ALS), based on a six exome sequencing sample data (three ALS samples B340, B350 and B360 and three normal samples B270, B310, B330). The results identified a novel Cys146X mutation of SOD1 that is highly correlated with ALS [31]. ALS, also called Lou Gehrig’s disease, is a progressive neurodegenerative disease characterized by progressive degeneration of the motor cells in the spinal cord and brain (central nervous system) [32]. To compare the performance of Exome-assistant with other similar tools, we used the multiple cases module of Exome-assistant, ANNOVAR and VAAST to perform a comparative analysis on the ALS sample SNPs/InDel data (28917/5733, 38232/5313 37216/5142, 39142/6115, 37444/4875 and 38113/5300 SNPs/InDels for B340, B350, B360, B270, B310 and B330, respectively). Firstly, the SNPs/InDels detected in the samples were annotated. Then, the missense/nonsense/splice site novel SNPs and novel InDels in coding region were picked out. Homozygous sites and the variations that were not significantly different within the CHB population in HAPMAP CHB were filtered out.

As results, Exome-assistant obtained 711/36, 623/37 and 837/36 candidate SNPs/InDels in three ALS samples B340, B350 and B360, respectively. 129/5 candidate SNPs/InDels were shared by three ALS samples, among which 29/2 SNPs/InDels were novel and contained the reported disease-related SOD1 Cys147X variation. Meanwhile, ANNOVAR obtained 419/46, 438/43, and 513/52 candidate SNPs/InDels in the three ALS samples, respectively, and 88/5 were shared by three ALS samples, in which the SOD1 gene variation was also contained in the 12/1 novel shared SNPs/InDels. VAAST obtained 687/36, 604/37 and 799/36 candidate SNPs/InDels in the three ALS samples, and 90/5 were shared by three samples. In the annotated 50/0 novel shared SNPs/InDels, the SOD1 gene variation was also identified. The results supported the efficiency of Exome-assistant compared with other widely-used tools.

## Conclusions

Recent technological advances in next-generation sequencing have greatly benefited studies on genetic variation. Exome sequencing offers a cost effective method for comprehensively screening variations in complex samples. The analysis of these variations and the selection of those contributing to the phenotype, especially disease-related phenotypes, from a large amount of variation data present both challenges and promises. In this study, Exome-assistant was developed as a public resource to permit the annotation and analysis of genetic variants identified from exome sequencing studies.

The main purpose of Exome-assistant is to provide a deep insight into genetic variation events. Exome-assistant provides a flexible and easy-to-use framework for annotating the variation results from next-generation sequencing platforms. Exome-assistant enables users to leverage the throughput and accuracy of the analysis, while facilitating its translation into biologically and biomedical meaningful results. Currently, Exome-assistant only supports the human genome, additional genomes of interest will be added in the future. Exome-assistant is free for non-commercial use and will be updated regularly. We welcome feedback from the user community. In the future, annotations of structural variations will be incorporated into Exome-assistant, and we will try to develop an FTP module, which allows users with limited internet connections to submit their data. In addition, further reference databases and parameters will be added in the future version of Exome-assistant, which will enable the user to filter non-disease related SNPs/InDels sites as much as possible, thus making the identified sites much more reliable. In summary, we believe that Exome-assistant will provide the scientific community with an integrated tool to assist their research into potential genetic variations contributing to diseases, based on data generated from exome sequencing.

## Availability and requirements

Project name: Exome-assistant

Availability: http://122.228.158.106/exomeassistant

Operating system: Exome-assistant is a web server hosted on an Apache 2.0 HTTP server under Linux operating system. The server of Exome-assistant is equipped with four Quad-Core AMD processors (2.2 GHz each) and 32 GB of RAM.

Programming Language: The front-end is implemented in PHP language, while the back-end pipeline is implemented in Perl language.

License: None required

Any restrictions to use by non-academics: No

The web application is implemented independently of operating system and has been successfully tested with Microsoft Internet Explorer 8.0 and Firefox 2/3 (under different versions of Linux, Microsoft Windows and MacOS).

## Abbreviations

SNPs: Single nucleotide polymorphisms; InDels: Insertions and deletions; KEGG: Kyoto Encyclopedia of Genes and Genomes; GO: Gene ontology; ALS: Amyotrophic lateral sclerosis.

## Competing interests

The authors declare that they have no competing interests.

## Authors’ contributions

QL and EJS developed the Exome-assistant web server and analysis pipeline, and drafted the manuscript. QJM, XW and XFL participated in pipeline development. XYL participated in drafting the manuscript and made substantial contributions to the conception of the study. ZSS and JYW were involved in planning of experiments and headed the project. ZSS and JYW revised the final version of the manuscript. All authors read and approved the final manuscript.
